# Comprehensive Genetic Characterization of a Spanish Brugada Syndrome Cohort

**DOI:** 10.1371/journal.pone.0132888

**Published:** 2015-07-14

**Authors:** Elisabet Selga, Oscar Campuzano, Mel·lina Pinsach-Abuin, Alexandra Pérez-Serra, Irene Mademont-Soler, Helena Riuró, Ferran Picó, Mònica Coll, Anna Iglesias, Sara Pagans, Georgia Sarquella-Brugada, Paola Berne, Begoña Benito, Josep Brugada, José M. Porres, Matilde López Zea, Víctor Castro-Urda, Ignacio Fernández-Lozano, Ramon Brugada

**Affiliations:** 1 Cardiovascular Genetics Centre, Institut d’Investigació Biomèdica de Girona (IDIBGi), Girona, Spain and Medical School, Universitat de Girona (UdG), Girona, Spain; 2 Paediatric Arrhythmia Unit, Cardiology Department, Hospital Sant Joan de Déu, University of Barcelona, Barcelona, Spain; 3 Arrhythmia Unit, Hospital Clínic of Barcelona, University of Barcelona, Barcelona, Spain; 4 Arrhythmia Unit, Hospital Universitario Donostia, San Sebastian, Spain; 5 Pediatric Cardiology Service, Hospital Ramón y Cajal, Madrid, Spain; 6 Arrhythmia Unit, Hospital Puerta de Hierro, Madrid, Spain; 7 Hospital Josep Trueta, Girona, Spain; Johns Hopkins University, UNITED STATES

## Abstract

**Background:**

Brugada syndrome (BrS) is a rare genetic cardiac arrhythmia that can lead to sudden cardiac death in patients with a structurally normal heart. Genetic variations in *SCN5A* can be identified in approximately 20-25% of BrS cases. The aim of our work was to determine the spectrum and prevalence of genetic variations in a Spanish cohort diagnosed with BrS.

**Methodology/Principal Findings:**

We directly sequenced fourteen genes reported to be associated with BrS in 55 unrelated patients clinically diagnosed. Our genetic screening allowed the identification of 61 genetic variants. Of them, 20 potentially pathogenic variations were found in 18 of the 55 patients (32.7% of the patients, 83.3% males). Nineteen of them were located in *SCN5A*, and had either been previously reported as pathogenic variations or had a potentially pathogenic effect. Regarding the sequencing of the minority genes, we discovered a potentially pathogenic variation in *SCN2B* that was described to alter sodium current, and one nonsense variant of unknown significance in *RANGRF*. In addition, we also identified 40 single nucleotide variations which were either synonymous variants (four of them had not been reported yet) or common genetic variants. We next performed MLPA analysis of *SCN5A* for the 37 patients without an identified genetic variation, and no major rearrangements were detected. Additionally, we show that being at the 30-50 years range or exhibiting symptoms are factors for an increased potentially pathogenic variation discovery yield.

**Conclusions:**

In summary, the present study is the first comprehensive genetic evaluation of 14 BrS-susceptibility genes and MLPA of *SCN5A* in a Spanish BrS cohort. The mean pathogenic variation discovery yield is higher than that described for other European BrS cohorts (32.7% vs 20-25%, respectively), and is even higher for patients in the 30-50 years age range.

## Introduction

Brugada syndrome (BrS) was identified as a new clinical entity in 1992 [1]. Six years later, the first genetic basis for the disease was identified, with the discovery of genetic variations in *SCN5A* [2]. Nowadays, more than 300 pathogenic variations in this first gene are known to be associated with BrS [3]. *SCN5A* encodes for the α subunit of the cardiac voltage-dependent sodium channel (Na_v_1.5), which is responsible for inward sodium current (*I*_Na_), and thus plays an essential role in phase 0 of the cardiac action potential (AP). Genetic variations in this gene can explain around 20–25% of BrS cases [3].

Since BrS was classified as a genetic disease, several other genes have been described to confer BrS-susceptibility [4–7]. Pathogenic variations have been mainly described in: 1) genes encoding proteins that modulate Na_v_1.5 function, and 2) other calcium and potassium channels and their regulatory subunits. All these proteins participate, either directly or indirectly, in the development of the cardiac AP. Although the incidence of pathogenic variations in these BrS-associated genes is low [6], it is considered that, among all of them, they could provide a genetic diagnosis for up to an extra 5–10% of BrS cases. Hence, altogether, a genetic diagnosis can be achieved approximately in 35% of clinically diagnosed BrS patients.

Other types of genetic abnormalities have been suggested to explain the remaining percentage of undiagnosed patients. Indeed, multiplex ligation-dependent probe amplification (MLPA) has allowed the detection of large-scale gene rearrangements involving one or several exons of *SCN5A* in BrS cases. However, the low proportion of BrS patients carrying large genetic imbalances identified to date suggests that this type of rearrangements will provide a genetic diagnosis for a modest percentage of BrS cases [8–10].

BrS has been associated with an increased risk of sudden cardiac death (SCD), despite the reported variability in disease penetrance and expressivity [11]. The prevalence of BrS is estimated at about 1.34 cases per 100 000 individuals per year, with a higher incidence in Asia than in the United States and Europe [12]. However, the dynamic nature of the typical electrocardiogram (ECG) and the fact that it is often concealed, hinder the diagnosis of BrS. Therefore, an exhaustive genetic testing and subsequent family screening may prove to be crucial in identifying silent carriers. A large percentage of these pathogenic variation carriers are clinically asymptomatic, and may be at risk of SCD, which is, sometimes, the first manifestation of the disease [13].

In the present work, we aimed to determine the spectrum and prevalence of genetic variations in BrS-susceptibility genes in a Spanish cohort diagnosed with BrS, and to identify variation carriers among relatives, which would enable the adoption of preventive measures to avoid SCD in their families.

## Results

### Study population

Overall, 55 unrelated Spanish patients clinically diagnosed with BrS were included in our study. Table 1 shows the demographics of this cohort, and Table 2 and S1 Table show the clinical and genetic characteristics of all the patients included in the study. The mean age at clinical diagnosis was of 41.9±13.3 years. Although the majority of patients were males (74.5%), their age at diagnosis was not different than that of females (41.8±12.1 years and 42.3±16.3 years, respectively; *p* = 0.92). A type 1 BrS ECG was present spontaneously in 37 patients (67.3%), and drug challenge revealed a type 1 BrS ECG for the remaining 18 patients (32.7%). Almost half of the patients had experienced symptoms, including 2 SCD and 4 aborted SCD. Patients who had not previously experienced any signs of arrhythmogenicity despite having a BrS ECG were considered asymptomatic. Comparison of symptomatic *vs* asymptomatic patients evidenced a similar percentage of males (73.1% and 75.9%, respectively). However, the mean age at diagnosis was different between the two groups of patients (37.7±14.3 and 45.7±11.4, respectively; *p*<0.05).

**Table 1:** Demographics of the 55 Spanish BrS patients included in the study. The table shows the demographic characteristics of all the patients included in the study. Numbers in parentheses represent the relative percentages for each condition. T1 ECG refers to Type 1 BrS diagnostic electrocardiogram (ECG), obtained either spontaneously, or after drug challenge. The information regarding both the electrophysiological studies (EPS) and the treatment was not available for all the patients. Two of the patients that didn’t receive any treatment died, and were not taken into account for the calculations of percentages (+2 dead). ICD, intracardiac cardioverter defibrillator.

Characteristic/Clinical presentation	Overall	Symptomatic Patients	Asymptomatic Patients
Number of Probands	55	26 (47.3%)	29 (52.7%)
Age at diagnosis, years	41.9±13.3; range 5–68	37.7±14.3; range 5–63	45.7±11.4; range 24–68
Males	41 (74.5%)	19/26 (73.1%)	22/29 (75.9%)
Females	14 (25.5%)	7/26 (26.9%)	7/29 (24.1%)
T1 ECG—spontaneous	37 (67.3%)	20/26 (76.9%)	17/29 (58.6%)
T1 ECG—drug challenge	18 (32.7%)	6/26 (23.1%)	12/29 (41.4%)
Family history of BrS	25 (45.5%)	11/26 (42.3%)	14/29 (48.3%)
Positive EPS	18/45 (40%)	8/18 (44.4%)	10/27 (37%)
Negative EPS	27/45 (60%)	10/18 (55.6%)	17/27 (63%)
ICD	32/46 (69.6%)	20/23 (87%)	12/23 (52.2%)
No treatment	12/46 (26.1%); +2 dead	1/23 (4.3%); +2 dead	11/23 (47.8%)

**Table 2:** Characteristics of the Spanish BrS patients carrying rare genetic variations. The table shows the clinical characteristics of the probands who carried rare genetic variations in *SCN5A*, *SCN2B*, or *RANGRF*. All of them are potentially pathogenic except that found in *RANGRF*, which is of unknown significance (see discussion). All the potentially pathogenic variations (PPVs) that had been previously reported, except p.P1725L and p.R1898C, had been identified in BrS patients. p.P1725L had been associated with Long QT Syndrome and p.R1898C was found in Exome Variant Server with a MAF of 0.0079%. No rare variations were identified in the control population. Patient’s age is expressed in years. Bold identifies the patients carrying variations that had not been described previously. M, male; F, female; S, syncope; ICD, intracardiac cardioverter defibrillator; UK, unknown; EPS, electrophysiological studies (+, positive response;-, negative response; N/P, not performed). The two patients who carried two PPVs each are identified by ^a^ and ^b^, respectively.

Proband Age/sex	Baseline ECG	Symptoms	ICD	EPS	Family historySCD	Family historyBrS	Gene	Aminoacidic change	Nucleotidic change	Reference
28/M	Type 1	S	Yes	+	No	Yes	*SCN5A*	p.R121W	c.361C>T	[14,15]
36/F	Type 1	S	UK	UK	No	No	*SCN5A*	p.R222*	c.664C>T	[3,16]
44/Ma	Type 1	None	Yes	+	No	No	*SCN5A*	p.P336L	c.1007C>T	[17]
45/M	Type 1	S	Yes	-	No	No	*SCN5A*	p.D356N	c.1066G>A	[18]
37/F	Type 1	S	Yes	-	Yes	Yes	*SCN5A*	p.R367H	c.1100G>A	[19–21]
33/M	Type 1	None	Yes	-	No	Yes	*SCN5A*	p.G386R	c.1156G>A	[3]
**41/M**	**Type 3**	**None**	**No**	**-**	**No**	**No**	***SCN5A***	p.R569Pfs*151	**c.1705dupC**	**Not reported**
8/M	Type 1	S	Yes	N/P	No	Yes	*SCN5A*	p.Q573*	c.1717C>T	[3]
**51/M**	**Type 1**	**None**	**No**	**-**	**Yes**	**Yes**	***SCN5A***	p.E625Rfs*95	**c.1872dupA**	**Not reported**
31/M	Type 1	S	Yes	-	No	Yes	*SCN5A*	p.I890T	c.2669T>C	[22]
49/M	Type 2	None	No	-	No	Yes	*SCN5A*	p.S910L	c.2729C>T	[23]
43/M	Type 1	None	Yes	+	No	Yes	*SCN5A*	p.R1232W	c.3694C>T	[2,24]
48/M	Type 2	S	Yes	+	No	Yes	*SCN5A*	p.D1243N	c.3727G>A	[3]
38/M^b^	Type 1	None	Yes	-	No	Yes	*SCN5A*	intronic	c.3840+1G>A	[3]
**31/M**	**Type 1**	**S**	**Yes**	**N/P**	**No**	**No**	***SCN5A***	p.R1623Efs*7	**c.4867delC**	**Not reported**
44/M^a^	Type 1	None	Yes	+	No	No	*SCN5A*	p.I1660V	c.4978A>G	[17,25]
38/M^b^	Type 1	None	Yes	-	No	Yes	*SCN5A*	p.D1690N	c.5068G>A	[26]
38/M	Type 1	S	Yes	-	No	No	*SCN5A*	p.P1725L	c.5174C>T	[27]
47/M	Type 3	S	Yes	+	No	No	*SCN5A*	p.R1898C	c.5692C>T	[28]
47/F	Type 1	S	Yes	+	No	No	*SCN2B*	p.D211G	c.632A>G	[7]
42/M	Type 2	None	Yes	+	Yes	Yes	*RANGRF*	p.E61*	c.181G>T	[29]

### Sequencing of genes associated with BrS

We performed a genetic screening of 14 genes (*SCN5A*, *CACNA1C*, *CACNB2*, *GPD1L*, *SCN1B*, *SCN2B*, *SCN3B*, *SCN4B*, *KCNE3*, *RANGRF*, *HCN4*, *KCNJ8*, *KCND3*, and *KCNE1L*), which allowed the identification of 61 genetic variations in our cohort. Of these, 20 were classified as potentially pathogenic variations (PPVs), one variation of unknown significance, and 40 common or synonymous variants considered benign.

The 20 PPVs were found in 18 of the 55 patients (32.7% of the patients, 83.3% males; Table 2). Sixteen patients (88.9%) carried one PPV, and two patients (11.1%) carried two different PPVs each. Nineteen out of the 20 PPVs identified were localized in *SCN5A* and one in *SCN2B*.

The vast majority of the PPVs identified were missense (70%). We also detected 2 nonsense variations (10%), 3 insertions or deletions causing frameshifts (15%), and one splicing variation (5%). The three frameshifts (p.R569Pfs*151, p.E625Rfs*95 and p.R1623Efs*7) were identified in *SCN5A*. These were not found in any of the databases consulted (see Methods), and were thus considered potentially pathogenic (see below). The other 16 rare variations identified in *SCN5A* had been previously described, and hence were also considered potentially pathogenic. Fourteen of them had been identified in BrS patients. Of these, 6 had also been identified in individuals diagnosed with other cardiac electric diseases (i.e. Sick Sinus Syndrome, Long QT Syndrome, Sudden Unexplained Nocturnal Death Syndrome or Idiopathic Ventricular Fibrillation [2,15,16,20,21,25]). The other 2, p.P1725L and p.R1898C, had only been associated with Long QT Syndrome or found in Exome Variant Server with a MAF of 0.0079%, respectively. Furthermore, we identified a variation in *SCN2B* (c.632A>G in exon 4 of the gene, resulting in p.D211G) which was considered pathogenic. This patient was included within our cohort, but the functional characterization of channels expressing *SCN2B* p.D211G was object of a previous study from our group [7]. We also identified a nonsense variation in *RANGRF* which has been formerly reported as rare genetic variation of unknown significance [29].

Additionally, we screened the relatives of those probands carrying a PPV. We analysed a total of 129 relatives, 69 of which (53.5%) were variation carriers. Genotype-phenotype correlations evidenced that 8 of the families displayed complete penetrance (S3 Table). Additionally, no relatives were available for one of the probands carrying a PPV, thus hampering genotype-phenotype correlation assessment. The other 12 families showed incomplete penetrance.

### MLPA analysis

The 37 patients with negative results after the genetic screening of the 14 BrS-associated genes underwent MLPA analyses of *SCN5A*. This technique did not reveal any large exon deletion or duplication in this gene for any of the patients.

### *SCN5A* p.R569Pfs*151 (c.1705dupC), a novel PPV

**Fig 1:**
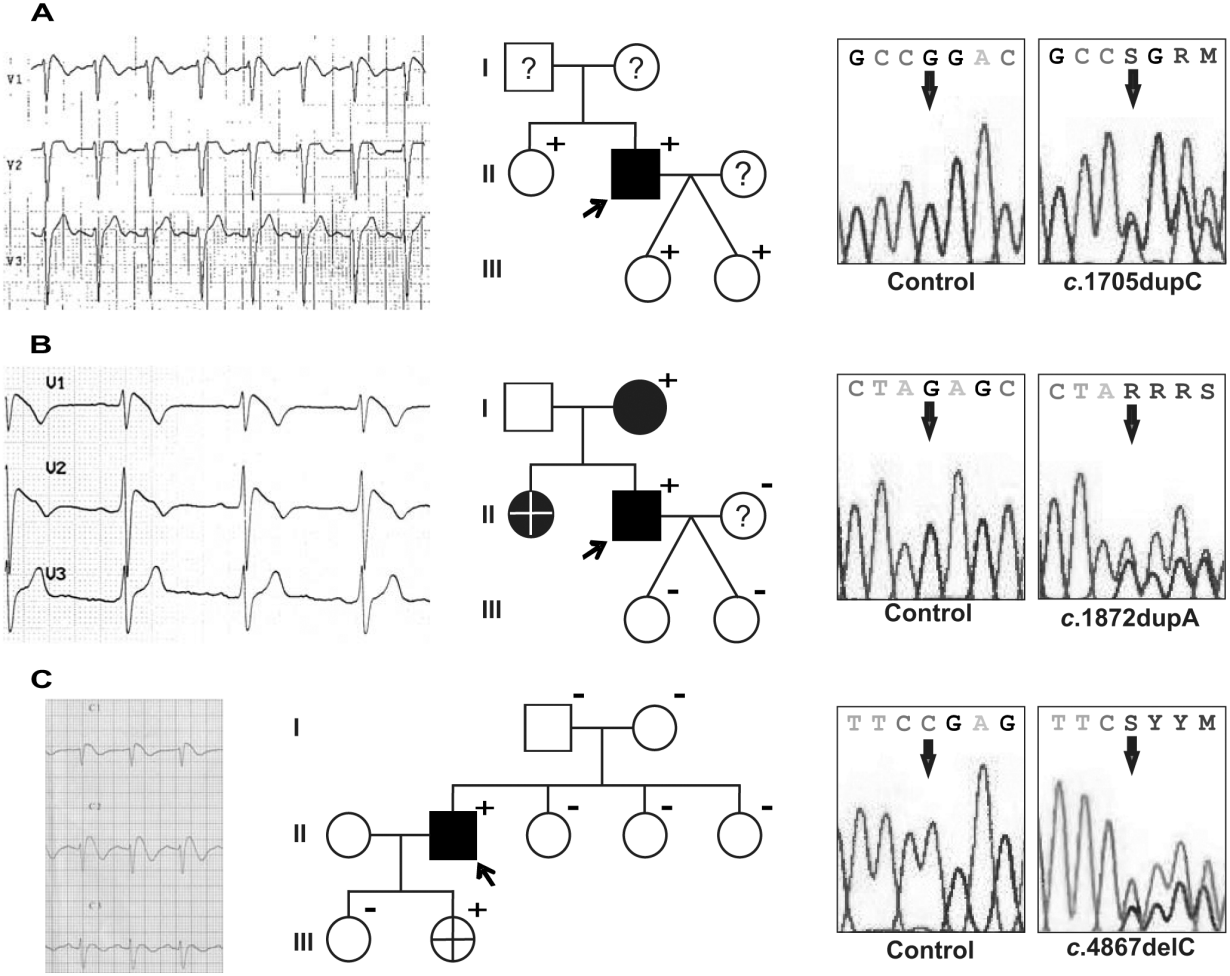
Characteristics of the probands carrying non-reported potentially pathogenic variations (PPVs) in *SCN5A* and their families. *Left*: Electrocardiograms of the probands: **(A)** patient carrying the p.R569Pfs*151 variation, showing the ST elevation characteristic of BrS in V1 at the time of the flecainide test; **(B)** patient carrying the p.E625Rfs*95 variation, showing the spontaneous ST elevation characteristic of BrS in V1 and V2; and **(C)** patient carrying the p.R1623Efs*7 variation, showing the spontaneous ST elevation characteristic of BrS in V1 and V2. *Middle*: Family pedigrees. Open symbols designate clinically normal subjects, filled symbols mark clinically affected individuals and question marks identify subjects without an available clinical diagnosis. Plus signs indicate the carriers of the PPVs and minus signs, non-carriers. The crosses mark deceased individuals and arrows identify the proband. *Right*: Detail of the electropherograms obtained after *SCN5A* sequence analysis of a control subject (left panels) and of the probands (right panels).

A 41-year-old asymptomatic male presented a type 3 BrS ECG which was suggestive of BrS. Flecainide challenge unmasked a type 1 BrS ECG (Fig 1A, left), which was also spontaneously observed sometimes during medical follow up. Sequencing of *SCN5A* revealed a duplication of a cytosine at position 1705 (c.1705dupC; Fig 1A, right), which originated a frameshift that lead to a truncated Na_v_1.5 channel (p.R569Pfs*151). The proband’s sister also carried this duplication, but had never presented signs of arrhythmogenesis. The proband’s twin daughters were also variation carriers, displayed normal ECGs and, to date, are asymptomatic (Fig 1A, middle). Thus, p.R569Pfs*151 represents a novel genetic alteration in the Na_v_1.5 channel that could potentially lead to BrS, but with incomplete penetrance.

### *SCN5A* p.E625Rfs*95 (c.1872dupA), a novel PPV

A 51-year-old asymptomatic male was diagnosed with BrS since he presented a spontaneous ST segment elevation in leads V1 and V2 characteristic of type 1 BrS ECG (Fig 1B, left). The sequencing of *SCN5A* evidenced an adenine duplication at position 1872 (c.1872dupA, Fig 1B, right). This genetic variation results in a truncated Na_v_1.5 channel (p.E625Rfs*95). The genetic analysis of the proband’s relatives proved that only her mother carried the variation (Fig 1B, middle). She was asymptomatic, but a BrS ECG was unmasked upon ajmaline challenge. The proband’s sister was found dead in her crib at 6 months of age, which suggests that her death might be compatible with BrS. Therefore, the p.E625Rfs*95 variation in the Na_v_1.5 channel represents a novel genetic alteration potentially causing BrS.

### *SCN5A* p.R1623Efs*7 (c.4867delC), a novel PPV

The proband, a 31-year-old male, was admitted to hospital after suffering a syncope. His baseline 12-lead ECG showed a ST segment elevation in leads V1 and V2 that strongly suggested BrS type 1 (Fig 1C, left). A deletion of the cytosine at position 4867 (c.4867delC) was observed upon *SCN5A* sequencing (Fig 1C, right). This base deletion leads to a frameshift that originates a truncated Na_v_1.5 channel (p.R1623Efs*7). Genetic screening of his parents and sisters evidenced that none of them carried this novel variation (Fig 1C, middle). None of them had presented any signs of arrhythmogenicity, nor had a BrS ECG. Nevertheless, *in utero* genetic analysis of one of his daughters proved that she had inherited the variation. She died when she was 1 year of age of non-arrhythmogenic causes. Hence, the p.R1623Efs*7 variation in the Na_v_1.5 channel is a novel genetic alteration originated *de novo* in the proband that could potentially lead to BrS.

### Synonymous and common genetic variations portrayal

In our cohort, we identified 40 single nucleotide variations which were common genetic variants and/or synonymous variants (S2 Table). Twenty-nine had a minor allele frequency (MAF) over 1%, and were thus considered common genetic variants.

We also identified 11 variants with MAF less than 1%. Of them, 9 were synonymous variants, what made us assume that they were not disease-causing. Four of these synonymous variants were not found in any of the databases consulted, and thus their MAF was considered to be less than 1%. Each of these synonymous variations was identified in 1 patient of the cohort. A similar proportion of individuals carrying these novel variations was detected upon sequencing of 300 healthy Spanish individuals (600 alleles). The remaining 2 variants were missense, and although they had either a MAF of less than 1% or an unknown MAF according to the Exome Variant Server and dbSNP websites, they were common in our cohort (29.2 and 50%, respectively; S2 Table), and a similar MAF was detected in a Spanish cohort of healthy individuals (26.7% and 48.8%, respectively).

### Influence of phenotype and age on PPV discovery

**Fig 2:**
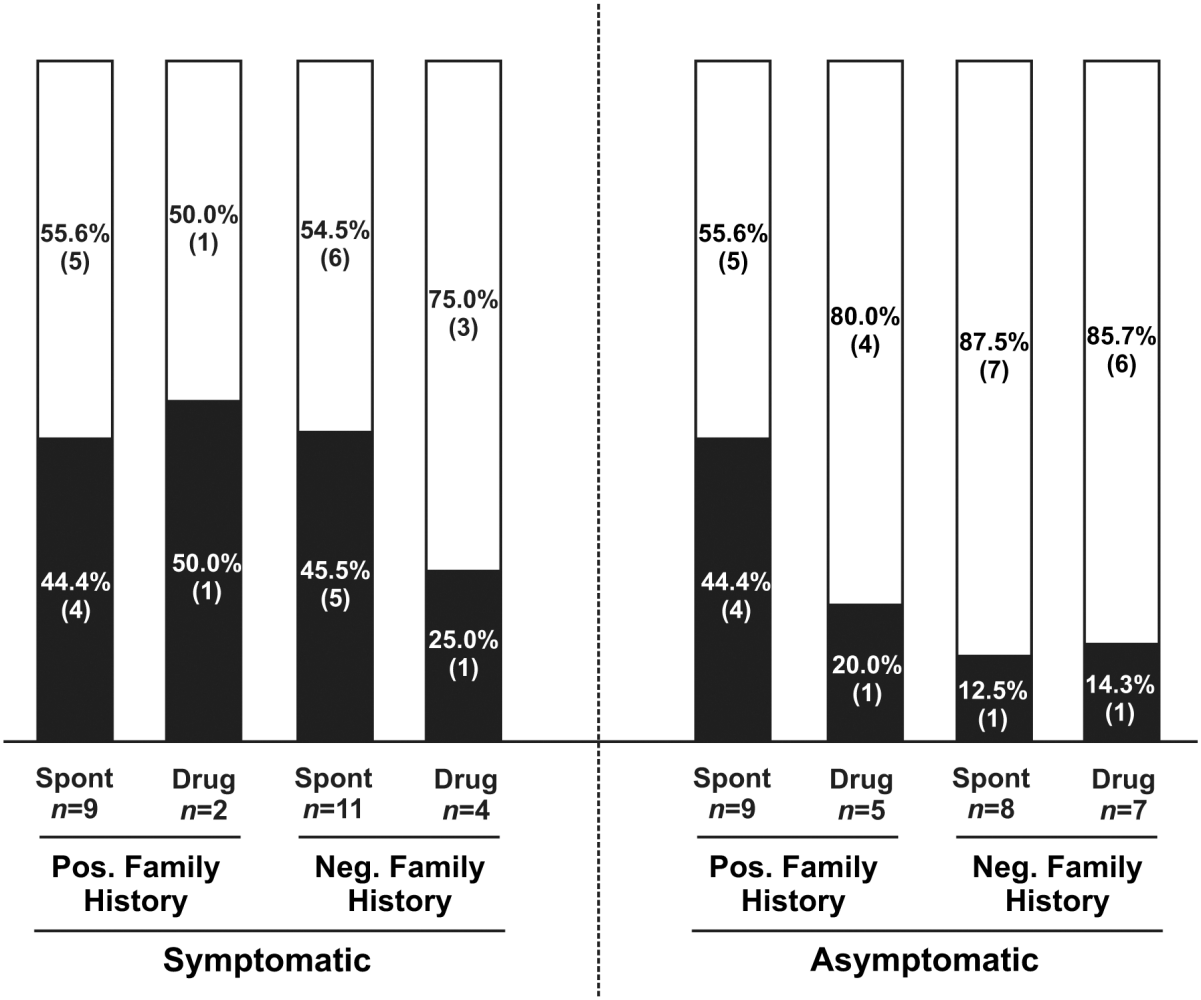
Influence of the phenotype on PPV discovery yield. Bar graph comparing the PPV detection yield in 8 different clinical categories (stated below the graph). Each bar shows the total number of patients for each clinical category divided in those with a PPV (black) and those without an identified PPV (white). The number of patients (in brackets) and percentages are given. Pos, positive; Neg, negative; Spont, spontaneous type 1 BrS ECG; Drug, drug-induced type 1 BrS ECG; *n*, number of patients.

To assess if a connection existed between the probands’ phenotype and the PPV detection yield, we classified the patients in our cohort according to their ECG (spontaneous or induced type 1), the presence of BrS cases within their families, and the presence/absence of symptoms. Even though the overall PPV detection yield was 32.7%, it was even higher for symptomatic patients (Fig 2). Indeed, in this group of patients, having a family history of BrS was identified as a factor for increased PPV discovery yield. In the case of absence of BrS in the family, the variation discovery yield was almost double for those patients having a spontaneous type 1 BrS ECG than for patients with drug-induced type 1 ECG (45.5% *vs* 25%, respectively). In addition, we identified a PPV in 44.4% of the asymptomatic patients who presented family history of BrS and a spontaneous type 1 BrS ECG. When the patient presented drug-induced type 1 ECG or in the absence of family history of BrS, the PPV discovery yield was of around 15%.

**Fig 3:**
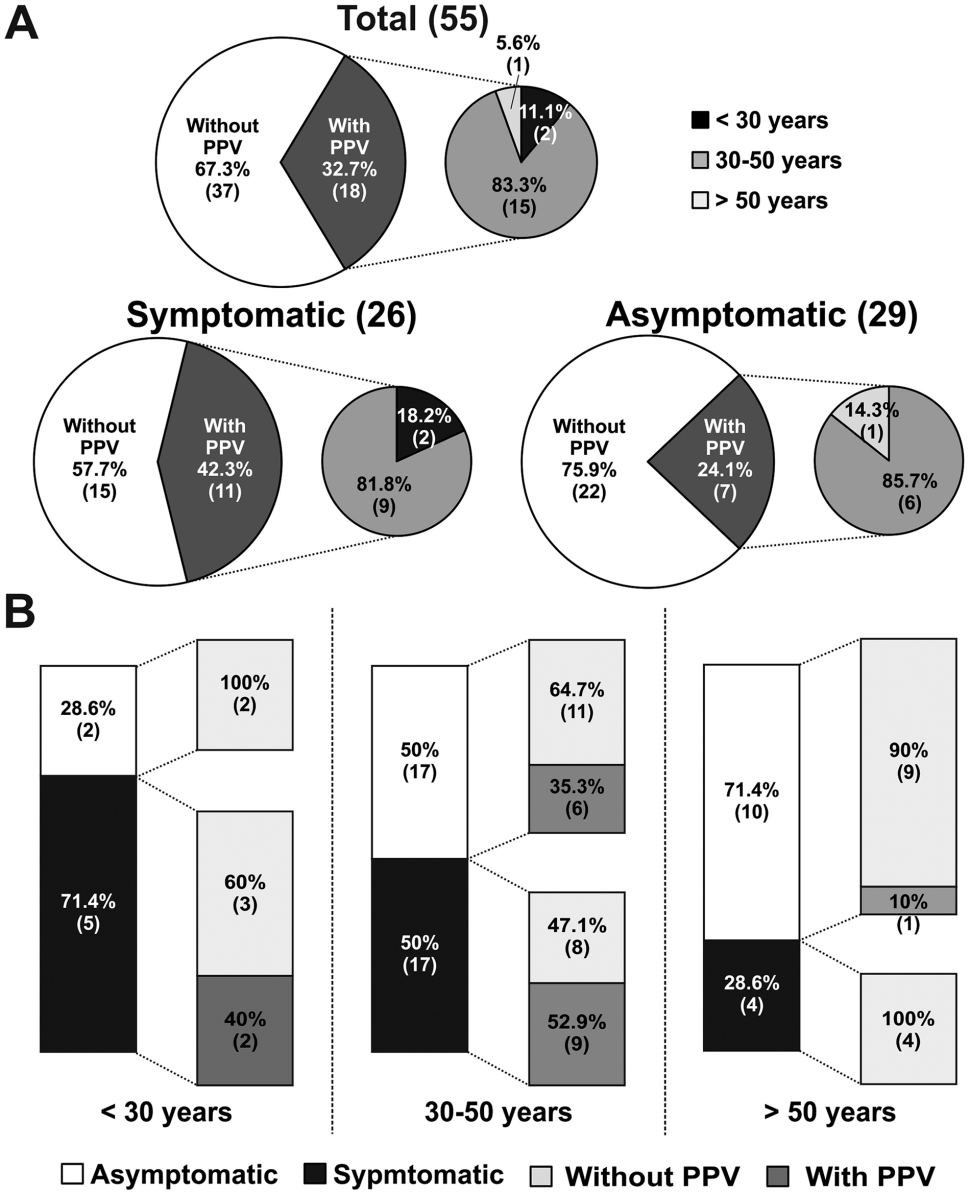
Influence of the age on PPVs discovery yield. **(A)** Pie charts showing the distribution of patients in the overall population as well as in the categories of symptomatic and asymptomatic patients regarding PPV discovery. The percentage and the number of patients (in brackets) are given for each group. The small pie charts correspond to the age distribution of patients with an identified PPV. **(B)** Bar graphs of the PPV detection yields obtained for each of the age groups (< 30 years, 30–50 years and > 50 years). Numbers inside each bar correspond to the number of patients carrying a PPV for each category and the percentages represent the variation detection yield.

We also investigated the role of age on the PPV occurrence. No significant age differences were observed between variation carriers and non-carriers (38.6±10.3 and 43.5±14.4, respectively, *p* = 0.16). However, the PPV discovery yield was higher for patients with ages between 30 and 50 years: out of the total of patients carrying a PPV, 83.3% of the patients were in this age range, while 11.1% were younger and 5.6% were older patients (Fig 3A, upper panel). The PPV discovery yield was significantly higher for symptomatic than for asymptomatic patients (42.3% *vs* 24.1%, respectively; Fig 3A, lower panels).

Noteworthy, in the 30–50 age range, 52.9% (9/17) of the symptomatic patients and 35.3% (6/17) of asymptomatic patients carried one PPV (Fig 3B, middle). Additionally, 40% (2/5) of the symptomatic young patients (< 30 years) were variation carriers, while no PPVs were identified in asymptomatic patients within this age range.

## Discussion

To the best of our knowledge, this is the first comprehensive genetic evaluation of 14 BrS-susceptibility genes and MLPA of *SCN5A* in a Spanish cohort. Well delimited BrS cohorts from Japan, China, Greece and even Spain have been genetically studied [24,30–32]. Additionally, an international compendium of BrS genetic variations identified in more than 2100 unrelated patients from different countries was published in 2010 [3]. However, all these studies screened *SCN5A* exclusively. In 2012, Crotti *et al*. reported the spectrum and prevalence of genetic variations in 12 BrS-susceptibility genes in a BrS cohort [5]. However, this study included patients of different ethnicity. Here, we report the analysis of 14 genes which has been conducted on a well-defined BrS cohort of the same ethnicity.

Our results confirm that *SCN5A* is still the most prevalent gene associated with BrS. Indeed, *SCN5A*-mediated BrS in our cohort (30.9%) is higher than the proportion described in other European reports [3,23], where a potentially causative variation is identified in only 20–25% of BrS patients. The reason for this discrepancy is unclear but could point towards a higher prevalence of *SCN5A* PPVs in the Spanish population or to selection bias. Additionally, we identified a genetic variation in *SCN2B* (c.632A>G, which results in p.D211G). We have formerly published the comprehensive electrophysiological characterization of this variation, and showed that indeed this variation could be responsible of the phenotype of the patient, thus linking *SCN2B* with BrS for the first time [7]. Also, we identified a variation in *RANGRF*. This variation (c.181G>T leading to p.E61X) had been previously reported in a Danish atrial fibrillation cohort [33]. Surprisingly, the authors reported an incidence of 0.4% for this variation in the healthy Danish population, which brought into question its pathogenicity. Our finding of this variation in an asymptomatic patient displaying a type 2 BrS ECG also points toward considering it as a rare genetic variation with a potential modifier effect on the phenotype but not clearly responsible for the disease [29].

No PPVs were identified in the other genes tested. Certainly, it is well accepted that the contribution of these genes to the disease is minor, and thus should only be considered under special circumstances [13,34]. In addition, recent studies have questioned the causality of variations identified in some of these minority genes [35].

We also used the MLPA technique for the detection of large exon duplications and/or deletions in *SCN5A* in patients without PPVs, and no large rearrangements were identified. This is in accordance with previous reports, which revealed that such imbalances are uncommon [8–10].

**Fig 4:**
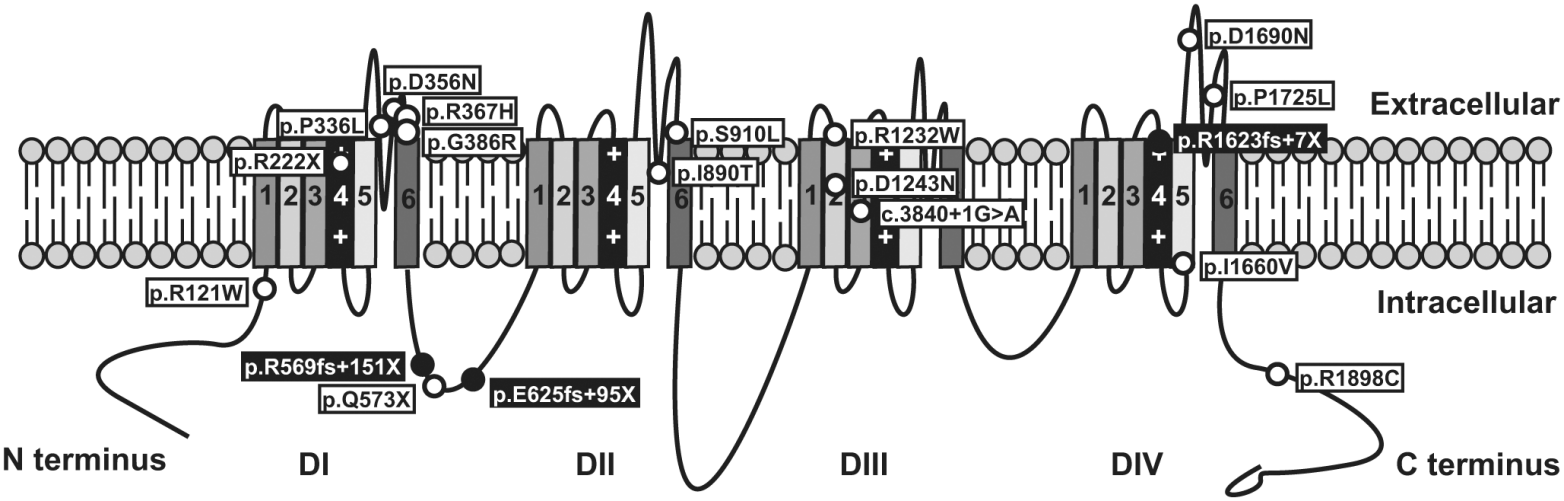
Na_v_1.5 channel scheme showing the relative position of the *SCN5A* PPVs identified in our cohort. Open symbols indicate already described variations and closed symbols locate novel variations reported in this study. DI to DIV designate the 4 domains of the protein, and numbers 1–6 identify the different segments within each domain. Crosses mark the voltage sensor.

Kapplinger *et al*. [3] reported a predominance of PPVs in transmembrane regions of Na_v_1.5. Indeed, it has been proposed that most rare genetic variations in interdomain linkers may be considered as non-pathogenic [36]. In contrast, PPVs identified in this study are mainly located in extracellular loops and cytosolic linker regions of Na_v_1.5 (Fig 4). Additionally, 2 of our non-previously reported frameshifts are located in the DI-DII linker. These 2 genetic variations lead to truncated proteins, which would lack around 75% of the protein sequence, and thus are presupposed to be pathogenic.

In our cohort, we have identified 40 synonymous or common genetic variations, 4 of which have not been previously reported. These variations are gradually becoming more and more important in the explanation of certain phenotypes of genetic diseases. Only a few common variations identified here are already published as phenotypic modifiers [37,38]. The effect of these and other common variants identified in our cohort on BrS phenotype should be further studied.

Unexpectedly, almost 40% (7/18) of the PPV carriers did not present signs of arrhythmogenicity. We also performed genotype-phenotype correlations of the PPVs identified in the families (S3 Table). These studies uncovered relatives, most of whom were young individuals, who carried a familial variation but had never exhibited any clinical manifestations of the disease. This is in agreement with Crotti *et al*. and Priori *et al*. [5,23], who postulated that a positive genetic testing result is not always associated with the presence of symptoms. Indeed, the existence of asymptomatic patients carrying genetic variations described to cause a severe Na_v_1.5 channel dysfunction has been reported [39]. The identification of silent carriers is of paramount importance since it allows the adoption of preventive measures before any lethal episode takes place. Unknown environmental factors, medication and modifier genes have been suggested to influence and/or predispose to arrhythmogenesis [11]. Hence, this group of patients has to be cautiously followed in order to avoid fatal events.

Our studies on the connection between patients’ phenotype and the PPV detection yield highlighted the presence of symptoms as a factor for an increased variation discovery yield. Within the group of symptomatic individuals, a PPV was identified in a higher proportion of patients displaying a spontaneous type 1 BrS ECG than for patients showing a drug-induced ECG. Likewise, within the asymptomatic patients with family history of BrS, those who presented spontaneous type 1 BrS ECG carried a PPV more often than those with a drug-induced ECG (Fig 2). Referring to age, the vast majority (17/20, 85%) of the PPVs were identified in patients around their fourth decade of age (30–50 years). This is in accordance with the accepted mean age of disease manifestation. Moreover, in this age range, more than 50% of the patients who presented symptoms carried a variation that could be pathogenic (Fig 3). Importantly, 35.3% of asymptomatic patients of around 40 years of age also carried one of such variations. These data highlight the importance of performing a genetic test even in the absence of clinical manifestations of the disease, and particularly when in the 30–50 years range, which is in accordance with consensus recommendations [13,34].

In conclusion, we have analysed for the first time 14 BrS-susceptibility genes and performed MLPA of *SCN5A* in a Spanish BrS cohort. Our cohort showed male prevalence with a mean age of disease manifestation around 40 years. BrS in this cohort was almost exclusively *SCN5A*-mediated. The mean PPV discovery yield in our Spanish BrS patients is higher than that described for other BrS cohorts (32.7% *vs* 20–25%, respectively), and is even higher for patients in the 30–50 years age range (up to 53% for symptomatic patients). All these evidences support the genetic testing, at least of *SCN5A*, in all clinically well diagnosed BrS patients.

## Study Limitations

First of all, drug challenge tests were not performed for all the relatives who were asymptomatic variation carriers. This fact hampered their clinical diagnosis and represents an impediment to definitely assess the link between PPVs and BrS. These patients are nowadays under follow-up.

New PPVs have been identified in our cohort. The clinical information available for the families suggests that these new variations could be pathogenic. Still, *in vitro* studies of these variations are required in order to evaluate their functional effects and verify their pathogenic role. Additionally, genotyping in an independent cohort would help reduce the likelihood of type I (false positive) error in genetic variant discovery.

We have to acknowledge that the study set is relatively small. Consequently, the classification of patients according to the different clinical categories rendered rather small sub-groups, which may lead to over-interpretation of the results. Future studies will be directed to the genetic screening of additional Spanish BrS patients, which will probably reinforce the significance of the tendencies observed here.

Also, the generally small size of the families limits the genotype-phenotype correlations performed. Incomplete penetrance could not be firmly assured in some families which include some young members that are PPV carriers. Although these individuals have not presented symptoms of BrS yet, they could be clinically diagnosed with BrS in the future. In addition, more family members should be studied to fully endorse complete penetrance in families with only one member currently diagnosed with the disease.

Finally, several BrS patients of our cohort carry no PPVs in any of the studied genes, but may carry genetic alterations either in the other genes that have been recently described to be associated with the disease or in other genes still to be associated with BrS. Furthermore, we cannot dismiss the presence of variations within gene regulatory regions, or the presence of large genomic rearrangements in genes other than *SCN5A*.

## Materials and Methods

### Patients

Spanish patients diagnosed with BrS were collected over the past 10 years. The clinical diagnosis was accepted as positive when the patients had a diagnostic (type 1) BrS ECG spontaneously or after the administration of intravenous sodium blockers, plus at least one of the following clinical criteria: occurrence of documented ventricular arrhythmia, family history of SCD or BrS, and/or symptoms secondary to arrhythmia [4]. Patient relatives were clinically diagnosed with BrS when they fulfilled the requirements stated above. All patients included in the genetic study had signed a written informed consent. The study complied with the requirements of the 1975 Declaration of Helsinki and was approved by the ethical committee of the institution (Hospital Josep Trueta, Girona, Spain).

### Sequencing of genes associated with BrS

Total genomic DNA was isolated from blood samples of the 55 patients and of 300 healthy Spanish individuals (individuals not related to any patient and of the same ethnicity; 600 alleles) using the Puregene DNA purification kit (Gentra Systems, Minneapolis, MI, USA). The genetic study was performed both in patients and in controls, and comprised the direct sequencing of *SCN5A* (NM_198056.2), *CACNA1C* (NM_001129827.1), *CACNB2* (NM_201596.2), *GPD1L* (NM_015141.3), *SCN1B* (NM_001037.4 for isoform a; and NM_199037.3 for isoform b), *SCN2B* (NM_004588.4), *SCN3B* (NM_018400.3), *SCN4B* (NM_174934.3), *KCNE3* (NM_005472.4), *RANGRF* (NM_001177801.1), *HCN4* (NM_005477.2), *KCNJ8* (NM_004982.3), *KCND3* (NM_004980.4), and *KCNE1L* (NM_012282.2) [40]. The exons and exon-intron boundaries of each gene were amplified (Verities PCR, Applied Biosystems, Austin, TX, USA), the PCR products were purified (Exosap-IT, Affymetrix, Inc. USB Products, Cleveland, OH, USA) and they were directly sequenced in both directions (Big Dye Terminator v3.1 cycle sequencing kit and 3130XL Genetic Analyzer, both from Applied Biosystems). The DNA sequences obtained were compared with their respective reference sequences (stated above). All variants detected were verified in an independent sequencing reaction from a new PCR product of the DNA of interest. The identified variations were compared with DNA sequences from the control patients, and contrasted with HGMD BioBase [41], HapMap [42], 1000 genomes project [40], NHLBI Exome Sequencing Project [28] and The Exome Aggregation Consortium (ExAC) [43]. Sequence changes altering coding regions were defined as genetic variations. Minor allele frequencies (MAFs) were checked in Exome Variant Server-NHLBI Exome Sequencing Project and dbSNP [44] databases. Genetic variations with a MAF in all populations <1% were considered rare variants. Genetic variations with a MAF >1% were considered common variants. Sequence variants were described following the HGVS rules [45], and checked in Mutalyzer [46]. All rare (MAF <1%) variants that had been previously described to be associated with BrS or other cardiac diseases were considered potentially pathogenic variations (PPVs). Stop and frameshift variants were always considered PPVs given their potential effects on ion channel function.

Samples were obtained for relatives of the patients who carried PPVs. DNA was extracted and it was screened for the presence of the PPV identified in the patients following the directions described above.

### Multiplex ligation-dependent probe amplification (MLPA) analysis of *SCN5A*

MLPA analysis was carried out in the 37 samples without an identified PPV using the commercially available SALSA MLPA P108 *SCN5A* probemix (MRC-Holland, Amsterdam, The Netherlands). This kit contains one probe for each exon of *SCN5A* and one probe upstream of the gene (isoform NM_198056.2). Remarkably, for exon 1 the probe is intronic, but very close to the exonic region, and for exon 28 two probes are included. The MLPA DNA detection and quantification were carried out according to the manufacturer’s protocol (MRC-Holland). After the multiplex PCR reaction, electrophoresis was performed using the ABI310 genetic analyzer with Liz 500 size standard (both from Applied Biosystems, CA, USA), and results were analysed using Coffalyser.Net (MRC-Holland). A reduction or increase in the relative signal strength of >30% was considered as a deletion or duplication of the locus, respectively.

### Statistics

Statistical comparisons were performed using the unpaired Student’s t-test in OriginPro 8. Results were considered statistically significant when *p*<0.05.

## Supporting Information

S1 Table Characteristics of the Spanish BrS patients with common and/or synonymous variations. (DOCX)

S2 Table Synonymous and common genetic variations in BrS-susceptibility genes identified in Spanish BrS patients. (DOCX)

S3 Table Genotype-phenotype correlation. (DOC)
