# Supplementary material for: Comprehensive Genetic Characterization of a Spanish Brugada Syndrome Cohort
Source: PLoS One. 2015 Jul 14;10(7):e0132888. doi: 10.1371/journal.pone.0132888 (PMC4501715; doi:10.1371/journal.pone.0132888)
Supplement: S1 Table — (DOCX) [file pone.0132888.s001.docx]

**S1** **Table.** **Characteristics of the Spanish BrS patients with common and/or synonymous variations.**

| **Proband Age/sex** | **Baseline ECG** | **Symptoms** | **ICD** | **EPS** | **Family history of BrS** |
| --- | --- | --- | --- | --- | --- |
| 32/M | Type 1 | aSCD | Yes | - | Yes |
| 54/M | Type 1 | None | UK | - | No |
| 46/F | Type 1 | S | Yes | + | No |
| 63/M | Type 1 | S | Yes | - | No |
| 43/M | Type 1 | aSCD | Yes | + | Yes |
| 45/M | Type 1 | aSCD | Yes | N/P | Yes |
| 13/M | Type 2 | SCD | Dead | N/P | No |
| 45/M | Type 1 | aSCD | No | - | No |
| 56/M | Type 2 | None | Yes | + | No |
| 57/M | Type 1 | S | Yes | - | No |
| 5/F | Type 1 | MVT | UK | UK | No |
| 64/M | Type 1 | None | No | - | No |
| 52/M | Type 2 | None | UK | - | No |
| 35/M | Type 1 | None | UK | - | No |
| 64/M | Type 1 | None | UK | + | Yes |
| 46/F | Type 1 | None | Yes | + | Yes |
| 40/M | Type 1 | None | Yes | - | No |
| 29/F | Type 2 | None | UK | - | Yes |
| 41/M | Type 1 | None | Yes | + | No |
| 35/M | Type 1 | SCD | Dead | N/P | No |
| 54/F | Type 1 | None | Yes | + | Yes |
| 34/M | Type 1 | None | No | UK | No |
| 68/F | Type 3 | None | No | UK | No |
| 30/M | Type 2 | Pre-S | Yes | + | Yes |
| 55/F | Type 1 | S | Yes | + | Yes |
| 30/F | Type 1 | None | No | - | Yes |
| 28/F | Normal | S | UK | - | No |
| 26/M | Type 3 | None | Yes | + | Yes |
| 55/M | Type 2 | S | Yes | N/P | No |
| 55/M | Type 2 | None | No | - | No |
| 44/M | Type 1 | None | Yes | + | Yes |
| 45/M | Type 1 | None | No | - | No |
| 33/M | Type 1 | S | Yes | - | Yes |
| 48/F | Type 2 | None | UK | - | Yes |
| 35/M | Type 3 | None | No | - | No |
| 63/F | Type 2 | None | No | - | No |

Table showing the clinical features of the patients without a genetic diagnosis. Their age is expressed in years. M, male; F, female; S, syncope; SCD, sudden cardiac death; aSCD, aborted SCD; MVT, Monomorphic ventricular tachycardia; UK, unknown; EPS, electrophysisological studies (+, positive response, -, negative response, N/P, not performed).
