# Supplementary material for: Comprehensive Genetic Characterization of a Spanish Brugada Syndrome Cohort
Source: PLoS One. 2015 Jul 14;10(7):e0132888. doi: 10.1371/journal.pone.0132888 (PMC4501715; doi:10.1371/journal.pone.0132888)
Supplement: S2 Table — (DOCX) [file pone.0132888.s002.docx]

**S2 Table.** **Synonymous and common genetic variations in BrS-susceptibility genes identified in Spanish BrS patients.**

| **Gene** | **Exon** | **Aminoacidic change** | **Nucleotidic change** | **Variation ID** | **MAF (%)** |
| --- | --- | --- | --- | --- | --- |
| *scn5a* | 2 | p.A29A | c.87A>G | [rs6599230](http://www.ncbi.nlm.nih.gov/SNP/snp_ref.cgi?rs=6599230) | 16.40 |
|  | **4** | **p.Q150Q** | **c.450G>A** | **Not reported** | **NR** |
|  | 12 | p.H558R | c.1673A>G | [rs1805124](http://www.ensembl.org/Homo_sapiens/Variation/Mappings?db=core;g=ENSG00000183873;r=3:38589548-38691164;t=ENST00000413689;v=rs1805124;vdb=variation;vf=17545788) | 24.63 |
|  | 17 | p.E1061E | c.3183A>G | [rs7430407](http://www.ensembl.org/Homo_sapiens/Variation/Mappings?db=core;g=ENSG00000183873;r=3:38589548-38691164;t=ENST00000413689;v=rs7430407;vdb=variation;vf=17745006) | 11.50 |
|  | 28 | p.D1819D | c.5457T>C | [rs1805126](http://www.ensembl.org/Homo_sapiens/Variation/Mappings?db=core;g=ENSG00000183873;r=3:38589548-38691164;t=ENST00000413689;v=rs1805126;vdb=variation;vf=17545790) | 43.14 |
| *cacna1c* | **3** | **p.T133T** | **c.399T>C** | **Not reported** | **NR** |
|  | 4 | p.A174A | c.522G>A | [rs1544514](http://www.ensembl.org/Homo_sapiens/Variation/Mappings?db=core;g=ENSG00000151067;r=12:2079952-2807115;t=ENST00000399655;v=rs1544514;vdb=variation;vf=21330076) | 17.57 |
|  | 17 | p.D812D | c.2436C>T | rs215976 | 7.91 |
|  | 30 | p.F1282F | c.3846C>T | [rs216008](http://www.ensembl.org/Homo_sapiens/Variation/Mappings?db=core;g=ENSG00000151067;r=12:2079952-2807115;t=ENST00000399655;v=rs216008;vdb=variation;vf=21285285) | 26.40 |
|  | 34 | p.I1394I | c.4182C>T | rs56180838 | 4.94 |
|  | **35** | **p.F1420F** | **c.4260C>T** | **Not reported** | **NR** |
|  | 44 | p.N1812N | c.5436C>T | [rs72552065](http://www.ensembl.org/Homo_sapiens/Variation/Mappings?db=core;g=ENSG00000151067;r=12:2079952-2807115;t=ENST00000399655;v=rs72552065;vdb=variation;vf=22052417) | 4.44 |
|  | 44 | p.T1835T | c.5505G>A | [rs1051375](http://www.ensembl.org/Homo_sapiens/Variation/Mappings?db=core;g=ENSG00000151067;r=12:2079952-2807115;t=ENST00000399655;v=rs1051375;vdb=variation;vf=21311680) | 41.89 |
|  | 46 | p.E1913K | c. 5737G>A | rs200231105 | 29.2* |
|  | 46 | p.T1918M | c.5753C>T | rs201777030 | 50* |
|  | 46 | p.P1931P | c.5793G>A | [rs56270948](http://www.ensembl.org/Homo_sapiens/Variation/Mappings?db=core;g=ENSG00000151067;r=12:2079952-2807115;t=ENST00000399655;v=rs56270948;vdb=variation;vf=21852297) | 8.30 |
| *cacnb2* | 13 | p.S452S | c.1365C>T | rs143060134 | 0.68 |
|  | 13 | p.Y567Y | c.1701C>T | [rs2228645](http://www.ensembl.org/Homo_sapiens/Variation/Mappings?db=core;g=ENSG00000165995;r=10:18429606-18830798;t=ENST00000396576;v=rs2228645;vdb=variation;vf=29472818) | 17.75 |
|  | 13 | p.D655E | c.1965T>G | [rs58225473](http://www.ensembl.org/Homo_sapiens/Variation/Mappings?db=core;g=ENSG00000165995;r=10:18429606-18830798;t=ENST00000396576;v=rs58225473;vdb=variation;vf=30057365) | 15.16 |
| *gpd1l* | 4 | p.D136D | c.408C>T | rs9835387 | 19.03 |
| *scn1b* | 4 | p.I167I | c.501T>C | [rs16969930](http://www.ensembl.org/Homo_sapiens/Variation/Mappings?db=core;g=ENSG00000105711;r=19:35521592-35531352;t=ENST00000262631;v=rs16969930;vdb=variation;vf=31195356) | 2.37 |
| *scn1bb* | 3 | **p.G196G** | **c.588G>A** | **Not reported** | **NR** |
|  | 3 | p.L210P | c.629T>C | rs55742440 | 42.14 |
|  | 3 | p.S248R | c.744C>A | rs67701503 | 12.48 |
|  | 3 | p.R250T | c.749G>C | rs67486287 | 11.64 |
| *SCN2B* | 2 | p.R47H | c.140G>A | rs17121818 | 1.30 |
| *scn3b* | 3 | p.A130A | c.390G>A | rs148484744 | 0.08 |
|  | 3 | p.T146T | c.438C>T | rs1275085 | 9.02 |
| *SCN4b* | 2 | p.C58C | c.174C>T | rs45539032 | 3.68 |
| *kcne3* | 1 | p.F66F | c.198T>C | [rs2270676](http://www.ensembl.org/Homo_sapiens/Variation/Summary?db=core;g=ENSG00000175538;r=11:74165886-74178600;t=ENST00000310128;v=rs2270676;vdb=variation;vf=14623887) | 11.41 |
|  | 1 | p.R83H | c.248G>A | rs17215437 | 1.10 |
| *hcn4* | 1 | p.L12L | c.36C>G | rs201193660 | 0.36 |
|  | 1 | p.G36E | c.107G>A | rs143090627 | 3.69 |
|  | 4 | p.L520L | c.1558C>T | [rs12909882](http://www.ensembl.org/Homo_sapiens/Variation/Mappings?db=core;g=ENSG00000138622;r=15:73612200-73661605;t=ENST00000261917;v=rs12909882;vdb=variation;vf=24044423) | 7.40 |
|  | 8 | p.V759I | c.2275G>A | [rs62641689](http://www.ensembl.org/Homo_sapiens/Variation/Mappings?db=core;g=ENSG00000138622;r=15:73612200-73661605;t=ENST00000261917;v=rs62641689;vdb=variation;vf=24235026) | 1.30 |
|  | 8 | p.P852P | c.2556G>A | [rs117819825](http://www.ensembl.org/Homo_sapiens/Variation/Mappings?db=core;g=ENSG00000138622;r=15:73612200-73661605;t=ENST00000261917;v=rs117819825;vdb=variation;vf=24722616) | 3.62 |
|  | 8 | p.A913A | c.2739G>A | rs373411041 | 0.07 |
|  | 8 | p.P1194P | c.3582A>G | TMP_ESP_15_73614852 | 0.02 |
| *kcnd3* | 1 | p.P88P | c.264C>T | [rs17221819](http://www.ensembl.org/Homo_sapiens/Variation/Mappings?db=core;g=ENSG00000171385;r=1:112313284-112531777;t=ENST00000315987;v=rs17221819;vdb=variation;vf=9149710) | 9.53 |
| *kcne1L* | 1 | p.P33S | c.97C>T | [rs17003955](http://www.ensembl.org/Homo_sapiens/Variation/Mappings?db=core;g=ENSG00000176076;r=X:108866929-108868393;t=ENST00000372101;v=rs17003955;vdb=variation;vf=16519683) | 9.68 |

The table compiles all the synonymous and common genetic variations that were identified in our cohort. The variation identifier (ID) is shown as it appears at the 1000 genomes browser database [27] or in NHLBI Exome Sequencing Project (ESP) Exome Variant Server (EVS) [30], and minor allele frequencies (MAFs) were obtained either from EVS website or from the dbSNP website [31]. * Frequency of these variations in our cohort. A similar MAF was detected in a Spanish cohort of healthy individuals (26.7% and 48.8%, respectively). MAF for p.E1913K in dbSNP was 0.8, and no data was available for p.T1918M in neither website. NR, not reported. Reference sequences were: *scn5a* (NM_198056.2), *cacna1c* (NM_001129827.1), *cacnb2* (NM_201596.2), *gpd1l* (NM_015141.3), *scn1b* (NM_001037.4 for isoform a; and NM_199037.3 for isoform b), *scn2b* (NM_004588.4), *scn3b* (NM_018400.3), *scn4b* (NM_174934.3), *kcne3* (NM_005472.4), *hcn4* (NM_005477.2), *kcnd3* (NM_004980.4) and *kcne1L* (NM_012282.2). Bold identifies the synonymous variants that had not been described previously. No genetic variations were identified in *KCNJ8*.
