# Supplementary material for: Comprehensive Genetic Characterization of a Spanish Brugada Syndrome Cohort
Source: PLoS One. 2015 Jul 14;10(7):e0132888. doi: 10.1371/journal.pone.0132888 (PMC4501715; doi:10.1371/journal.pone.0132888)
Supplement: S3 Table — (DOC) [file pone.0132888.s003.doc]

|  |  | **Symptomatic** | | **Asymptomatic** | |  |
| --- | --- | --- | --- | --- | --- | --- |
| **Gene** | **Aminoacidic change** | **Carriers** | **Non-carriers** | **Carriers** | **Non-carriers** | **Penetrance** |
| *SCN5A* | p.R121W | 7 (T1,  *n*=1; Inducible,  *n*=2; Syncope,  *n*=4) |  | 2 (young) | 7 | Incomplete^d^ |
| *SCN5A* | p.R222* | 1 (T1) | - | - | - | - |
| *SCN5A* | p.P336L^a^ | 2 (T1,  *n*=1; Inducible,  *n*=1) | - | - | 2 | Complete |
| *SCN5A* | p.D356N | 1(T1) | - | - | 2 | Complete^e^ |
| *SCN5A* | p.R367H | 15 (T1, *n*=10; Inducible,  *n*=5) | 2 (Inducible) | 2 | 7 | Incomplete |
| *SCN5A* | p.G386R | 3 (T1) | - | - | 5 | Complete |
| ***SCN5A*** | **p.R569Pfs*151** | 1 (Inducible) | - | 3 (2 young) | - | Incomplete^d^ |
| *SCN5A* | p.Q573* | 2 (T1) | - | - | 1 | Complete |
| ***SCN5A*** | **p.E625Rfs*95** | 2 (T1,  *n*=1; Inducible,  *n*=1) | - | - | 4 | Complete |
| *SCN5A* | p.I890T | 3 (T1,  *n*=1; Inducible,  *n*=1; Syncope,  *n*=1) | - | 1 (young) | 1 | Incomplete^d^ |
| *SCN5A* | p.S910L | 2 (Inducible) | - | - | 2 | Complete |
| *SCN5A* | p.R1232W | 3 (T1,  *n*=2; Inducible,  *n*=1) | - | 1 (young) | 1 | Incomplete^d^ |
| *SCN5A* | p.D1243N | 3 (Inducible) | - | - | 3 | Complete |
| *SCN5A* | intronic^b^ | 3 (T1,  *n*=1; Inducible,  *n*=1; Syncope,  *n*=1) | - | 1 (young) | 5 | Incomplete^d^ |
| ***SCN5A*** | **p.R1623Efs*7** | 1 (T1) | - | 1 (young) | 5 | Incomplete^d^ |
| *SCN5A* | p.I1660V^a^ | 1 (T1) | 1^c^ (Inducible) | 1 (young) | 1 | Incomplete^d^ |
| *SCN5A* | p.D1690N^b^ | 3 (T1,  *n*=1; Inducible,  *n*=1; NA,  *n*=1) | - | 1 (young) | 5 | Incomplete^d^ |
| *SCN5A* | p.P1725L | 1 (T1) | - | 1 (young) | 3 | Incomplete^d^ |
| *SCN5A* | p.R1898C | 1 (T3) | - | - | 1 | Complete^e^ |
| *SCN2B* | p.D211G | 1 (T1) | - | 3 | 6 | Incomplete |
| *RANGRF* | p.E61* | 3 (Inducible) | - | 2 (young) | 2 | Incomplete^d^ |

**S3 Table. Genotype-phenotype correlation.**

The table shows the classification of relatives from probands who carried rare genetic variations in *SCN5A*, *SCN2B*, or *RANGRF* according to their clinical and genetic status. Information regarding the electrocardiogram (ECG) type recorded for symptomatic family members is provided (T1, basal Type 1 BrS ECG; Inducible, Type 1 BrS ECG could be induced upon drug challenge; *n*, number of individuals). Six of the relatives had presented with syncope, but no information is available regarding their ECG. Also, a column specifying if penetrance is complete or incomplete in the family is provided. Bold identifies the patients carrying variations that had not been described previously. The two families with members carrying 2 PPVs are identified by ^a^ and ^b^, respectively. The individual identified with ^c^ is a member of one of these families, and only carries one of the 2 familiar PPVs (p.P336L), but displays symptoms of BrS. Incomplete penetrance cannot be firmly assured in families identified with ^d^ because they include some young members that are PPV carriers but, although they have not presented symptoms of BrS yet, they could be clinically diagnosed with BrS in the future. More family members should be studied to fully endorse complete penetrance in families identified with ^e^.
